# Communicating risk during early phases of COVID-19: Comparing governing structures for emergency risk communication across four contexts

**DOI:** 10.3389/fpubh.2023.1038989

**Published:** 2023-01-27

**Authors:** Brogan Geurts, Heide Weishaar, Almudena Mari Saez, Florin Cristea, Carlos Rocha, Kafayat Aminu, Melisa Mei Jin Tan, Bienvenu Salim Camara, Lansana Barry, Paul Thea, Johannes Boucsein, Thurid Bahr, Sameh Al-Awlaqi, Francisco Pozo-Martin, Evgeniya Boklage, Alexandre Delamou, Ayodele Samuel Jegede, Helena Legido-Quigley, Charbel El Bcheraoui

**Affiliations:** ^1^Evidence-Based Public Health Unit, Center for International Health Protection, Robert Koch Institute, Berlin, Germany; ^2^Center for International Health Protection, Robert Koch Institute, Berlin, Germany; ^3^Department of Sociology, Faculty of the Social Sciences, University of Ibadan, Ibadan, Nigeria; ^4^Saw Swee Hock School of Public Health, National University of Singapore, Singapore, Singapore; ^5^African Center of Excellence for the Prevention and Control of Communicable Diseases, Conakry, Guinea; ^6^Centre de Formation et de Recherche en Santé Rurale de Maferinyah, Département de Recherche, Unité de Socio-Anthropologie, Conakry, Guinea; ^7^Department of Infectious Disease Epidemiology, Robert Koch Institute, Berlin, Germany; ^8^Postgraduate Training for Applied Epidemiology, Robert Koch Institute, Berlin, Germany; ^9^European Programme for Intervention Epidemiology Training, European Centre for Disease Prevention and Control (ECDC), Stockholm, Sweden; ^10^Information Center for International Health, Center for International Health Protection, Robert Koch Institute, Berlin, Germany

**Keywords:** emergency risk communication, outbreak response, community engagement, international health regulations (IHR), public health emergencies, compliance, risk communication, health policy

## Abstract

**Background:**

Emergency risk communication (ERC) is key to achieving compliance with public health measures during pandemics. Yet, the factors that facilitated ERC during COVID-19 have not been analyzed. We compare ERC in the early stages of the pandemic across four socio-economic settings to identify how risk communication can be improved in public health emergencies (PHE).

**Methods:**

To map and assess the content, process, actors, and context of ERC in Germany, Guinea, Nigeria, and Singapore, we performed a qualitative document review, and thematically analyzed semi-structured key informant interviews with 155 stakeholders involved in ERC at national and sub-national levels. We applied Walt and Gilson's health policy triangle as a framework to structure the results.

**Results:**

We identified distinct ERC strategies in each of the four countries. Various actors, including governmental leads, experts, and organizations with close contact to the public, collaborated closely to implement ERC strategies. Early integration of ERC into preparedness and response plans, lessons from previous experiences, existing structures and networks, and clear leadership were identified as crucial for ensuring message clarity, consistency, relevance, and an efficient use of resources. Areas of improvement primarily included two-way communication, community engagement, and monitoring and evaluation. Countries with recurrent experiences of pandemics appeared to be more prepared and equipped to implement ERC strategies.

**Conclusion:**

We found that considerable potential exists for countries to improve communication during public health emergencies, particularly in the areas of bilateral communication and community engagement as well as monitoring and evaluation. Building adaptive structures and maintaining long-term relationships with at-risk communities reportedly facilitated suitable communication. The findings suggest considerable potential and transferable learning opportunities exist between countries in the global north and countries in the global south with experience of managing outbreaks.

## 1. Introduction

Communication about risks and preventive measures is key to managing public health emergencies (PHE) (1, 2). The aim of emergency risk communication (ERC) is to allow vulnerable individuals to make decisions to reduce the risks they are exposed to and protect themselves against infection (3). Due to the global scale, its dynamic nature, and the prevalence of social media, the COVID-19 pandemic has posed both new challenges and opportunities for ERC (4, 5). Studies which compare non-pharmaceutical interventions during the COVID-19 pandemic show that active communication with the public and coordinated public information campaigns significantly reduce the spread of COVID-19 (6, 7). Communication has further played a crucial role in promoting vaccination uptake and increasing vaccination readiness (8, 9). Yet, analyses of communication during the pandemic also indicate that ERC coordination was lacking, that the public's needs for information particularly in the early stages of the pandemic were not met, and communication on vaccination was, at best, mediocre (10–12).

A report on the functioning of the International Health Regulations during the COVID-19 response postulates that WHO and country capacities and approaches to information management and risk communication urgently need to be improved (13). While learning from past experiences of ERC is acknowledged as crucial, little is known about how ERC was actually designed and implemented in the context and early stages of COVID-19 (4). Existing studies focus on describing risk communication strategies and understanding the use of specific information sources, channels, target audiences, and messages (14, 15). A systematic review on integrating risk communication into emergency responses (16, 17) formed the basis of WHO evidence-based guidance for risk communication in PHE (3). These guidelines provide key recommendations for developing and implementing ERC, including building trust, integrating ERC into emergency response, and ERC practice. Yet, research which identifies good practice and analyses ways of improving ERC, particularly in low and middle income countries, are largely lacking (3). This knowledge gap means that important lessons for the improvement of ERC in future PHE may remain unseized (3).

In this study, we aim to investigate and compare ERC governing structures in the early stages of the COVID-19 pandemic across four socio-economic contexts, thereby identifying aspects of ERC that were perceived to be successful or requiring improvement. More specifically, we apply Walt and Gilson's health policy framework to identify key factors with regard to content, process, actors, and context that can support how ERC strategies are developed and implemented during PHE (18).

## 2. Methods

Qualitative research methods were employed to capture the dynamic nature of ERC within an ongoing pandemic. Qualitative methods were chosen as they were perceived as most suitable for obtaining an in-depth insight into how ERC was developed and implemented during the COVID pandemic, particularly given that data about respective processes is not routinely collected or possible to elicit *via* quantitative methods. Data were collected through a document review and key informant interviews in Germany, Guinea, Nigeria, and Singapore between June and December 2020. The four countries present a variety of economic and epidemiological contexts. According to the World Bank, Guinea is a low-income country, Nigeria a lower middle income country, and Germany and Singapore are high income countries (19). The countries also differ with regard to their epidemiological profile: by the 31st December 2020, the end of the data collection period, the cumulative incidence of COVID-19 cases per 100,000 had reached 40 in Nigeria, 99 in Guinea, 1039 in Singapore, and 2062 in Germany (20).

### 2.1. Document review

Following a scoping review of the literature, we performed a content analysis of documents collected through an online search of ERC-relevant websites and materials, including relevant ministries, public health bodies, civil society organizations, and COVID-19 specific campaigns. Media reports, non-country specific documents, and documents relating to secondary (e.g., economic) impacts of COVID-19 were excluded. Searches were restricted to a timeframe from 1 December 2019 when the first COVID-19 cases were reported to 31 July 2020 when fieldwork began. All relevant documents were analyzed using a content analysis framework and a set of pre-defined categories derived from the existing ERC literature.

### 2.2. Key informant interviews

Based on the results of the document review, an interview guide was developed covering the following categories: (1) organizations involved in the ERC strategy, (2) collaboration between organizations, (3) message design and implementation, (4) public and community engagement, and (5) sustainability. Key informants were identified through the document review and stakeholder mapping and were sampled using purposeful sampling and snowball methods at national and local levels.

Ethical approval was obtained from the ethics committee of the Charité–Universitätsmedizin Berlin (EA2/148/20), the Comité National D'Ethique pour la Recherche en Santé (105/CNERS/20) in Guinea, the National Health Research Ethics Committee of Nigeria (NHREC/01/01/2007-19/08/2020), and the National University Singapore Institutional Review Board (NUS-IRB-2020-434).

Fieldwork was conducted between August and December 2020. All interviews were conducted in an official language of the country or a local language either online or face-to-face in accordance with local COVID-19 prevention measures. All key informants were provided an information sheet and consent form prior to the interview and gave verbal and written consent. All interviews were audio-recorded, transcribed verbatim, professionally translated to English if necessary, and anonymised.

A coding scheme was developed based upon the topic guide, pre-existing ERC literature, and inductive co-coding of a subset of the data by the research team and then systematically applied to the entire data set. Interviews were analyzed using inductive and deductive thematic content analysis in NVIVO (21). Thematic saturation was achieved when ‘rich information’ was identified in each of the themes and no more relevant themes were emerging from the data.

Ethical approval was obtained from the ethics committees in each of the participating countries.

### 2.3. Patient and public involvement

Scoping interviews were conducted with key stakeholders involved in ERC in the early stages of the study design and topic guide development. As part of a larger study, focus group discussions (FGD) were conducted with the general public and select at-risk populations to illicit and understand perceptions and reception of ERC during the early phases of the COVID-19 pandemic. FGD and KII iteratively informed each other and refinement areas and served as triangulation points. FGD data are however not presented in the scope of this but in another article (22).

### 2.4. Analytical framework

This article draws on Walt and Gilson's health policy framework to analyse the content, process, actors, and context of how ERC strategies were developed and implemented during the early stages of the COVID-19 pandemic. Content refers to the substance of policies including their background, aims, and objectives; process comprises the actions which were undertaken to design and implement the policies; actors refers to key individuals, groups or organizations involved, including their roles and relationships within the design and implementation of policies; context encapsulates the socio-political, economic, historical, and cultural contexts within which policies are situated. The framework aims at systematically understanding the dynamics and interconnectedness of these aspects throughout the development and implementation of health policy (18). A 2020 review of studies that applied Walt and Gilson's framework shows that the framework has been used to analyse health policies at national, international and cross-country levels, including policies in high-, low- and middle-income countries. Several studies have applied Walt and Gilson's framework to triangulate different kinds of qualitative data (e.g., document review and key informant interviews) and develop an in-depth understanding and analyse the development and implementation of health policies, similarly to the approach that we pursued in this study. Applying the framework to cross-country comparisons was considered to help identify general, as well as, context-specific aspects of ERC (23).

## 3. Results

A total 142 semi-structured key informant interviews were conducted with 155 key stakeholders at national, regional, and local levels from various sectors involved in the design and implementation of ERC strategies (Table 1). In Singapore, interviews were only conducted at national level due to Singapore being a city-state.

**Table 1:** Summary of key informant interviews.

**Types of key informants**	**Germany** **(*n* = 56)**	**Guinea** **(*n* = 38)**	**Nigeria** **(*n* = 46)**	**Singapore** **(*n* = 15)**	**Total** **(*n* = 155)**
Academic	6	1		7	14 (9%)
Community representative	1	6	12		19 (12%)
Health care worker/professional	1	3	1		5 (3%)
International/intergovernmental organization		6	9		15 (10%)
Media	4	5	2		11 (7%)
Non-governmental organization	11	6	6	5	28 (18%)
Political decision maker	13	1			14 (9%)
Public health administration/authority	20	10	16	3	49 (32%)

### 3.1. Content

All countries have policies that outline ERC during pandemics (Supplement material), whose content and approach, however, differ considerably (Table 2). While Guinea and Nigeria published explicit COVID-19 risk communication and public engagement strategies in the spring of 2020, Germany and Singapore largely relied on strategies outlined in their existing or supplemental national pandemic plans. Having a designated ERC lead, defining priority audiences, including at-risk groups, specifying activities, and incorporating monitoring, rumor management and evaluation as an integral part into the strategy emerged as key features reported by key informants as contributing to clear and targeted communication.

**Table 2:** Comparison of ERC policies in study countries.

	**Germany (24–26)**	**Guinea (27)**	**Nigeria (28–31)**	**Singapore (32, 33)**
Policy document outlining ERC strategy	No specific document on ERC; ERC defined in the national pandemic plan I and II and the supplement to the national pandemic plan on COVID	Annual communication, social mobilization and community engagement plan for COVID-19 in Guinea	risk communication and community engagement strategy	No specific document on ERC; communication included in the ministry of health pandemic readiness and response plan for influenza and other acute respiratory diseases
Author	National public health institute (RKI)	Ministry of health, national health security agency (ANSS)	Presidential task force and Nigerian center for disease control	Ministry of health
Publication date	2016/2017, 2020 (supplement)	2020	2020	2014
Theoretical basis for ERC stated in policy	Not explicitly stated, however described in Part II of the National Pandemic Plan	Not stated	Extended Parallel Processing Model and Socio-ecological model	Not stated
Goal of ERC	Clearly stated: “to provide rapid, comprehensive and consistent information to all stakeholders and the public using all available media” (24) “…with the goal of minimizing the number of cases of illness and severe progressions of the disease in Germany” (25)	Clearly stated: “to ensure [multi-sectoral] commitment to concrete actions to break the chain of transmission of the virus” (27)	Clearly stated: “to provide frequent, timely and actionable information to empower individuals to take individual and collective responsibility to prevent and limit the spread of COVID-19, by practicing priority health behaviors that protect themselves and their communities” (30)	Clearly stated: “to communicate with and educate the public and securing their co-operation with our efforts” (33)
Priority audiences	Clearly defined (General public, governmental representatives, vulnerable groups, health professionals, media)	Clearly defined (General public, governmental representatives, schools, vulnerable groups, community leaders and mobilisers)	Clearly defined (General public, policy makers, community leaders, health professionals, policy makers)	Clearly defined (General public, policy makers, health professionals)
Rumor management	Necessity briefly stated	How-to described	How-to described, including detail on different phases and responsibilities	Not stated
Monitoring and evaluation	Not stated	Described, including responsibilities and methods	Described, including data collection tool and analysis plan, data flow, data use, feedback mechanism, and archiving	Not stated

### 3.2. Process

**Figure 1:**
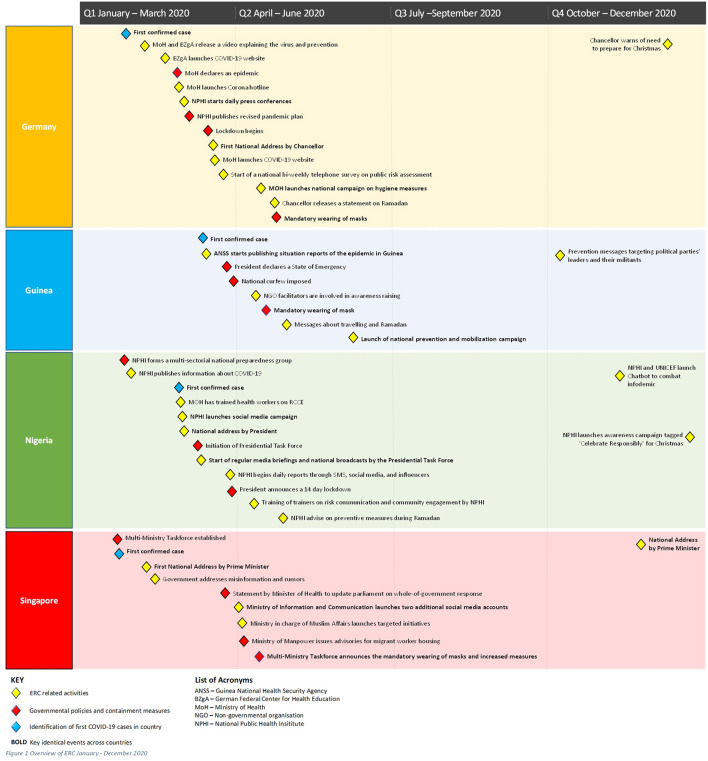
Overview of ERC January–December 2020.

An overview of the chronology of ERC across the four countries showed that the identification of the first COVID-19 case in each country triggered both governmental policies and containment measures including specific ERC activities (Figure 1).

The short time between the first outbreak in China and the local onset of COVID-19 and the high speed at which transmission occurred locally meant that there was little time to prepare and implement ERC strategies. The degree to which this time was seized to prepare ERC was perceived as crucial, as illustrated by a key informant in the Nigerian Ministry of Health:

“We were able to develop the risk communication and community engagement strategy [after hearing about the outbreak in China] and then we had not finalized before we had the first case in February […] After the first [local] case, we intensified the rate at which we were developing the strategy.” (NI 07)

The rapid development of the pandemic further required quick scale-up of activities, flexibility, and a need for country-wide ERC capacity-building and alignment. Interviewees reported that ERC was amended constantly in line with the evolving epidemiological situation, emerging evidence, changing policies, and developments in public reception and behavior. While public feedback and engagement were acknowledged to be crucial, respondents stressed that monitoring, evaluation, and subsequent message adaptation were difficult to implement simultaneously. When monitoring mechanisms were built into the ERC strategy as an integral component in Nigeria, this facilitated the use of feedback in the adaptation of messages. Monitoring in the form of surface-level recording of output numbers (e.g., social media clicks, number of posters distributed, volume of calls to hotlines) in other countries was perceived as superfluous. Respondents from Germany and Singapore further recalled that existing monitoring data, including social media data, were underutilized due to a lack of systematic and strategic evaluation mechanisms.

### 3.3. Actors

**Figure 2:**
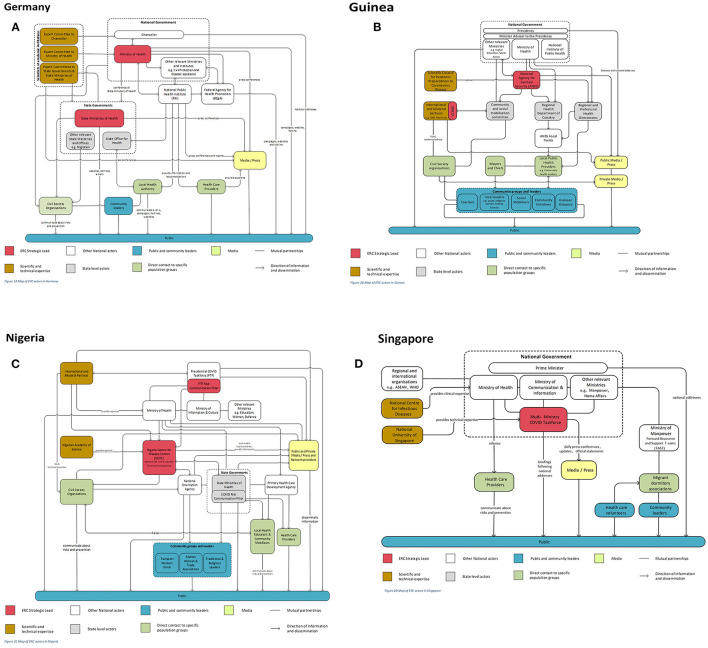
**(A–D)** Maps of main ERC actors involved in strategy development and implementation.

Figures 2A–D graphically depict the relationships between the main actors involved in the ERC strategy development and implementation in each country.

In all countries, a governmental actor (e.g., the national Ministry of Health, the national public health institute, or a multi-ministry taskforce) could be identified as leading ERC. Most respondents suggested that ERC functioned particularly efficiently *via* dedicated leads that were backed by cross-governmental support. A leader of a German research institute illustrated that “if there is one who communicates, then you have solved your problem with cacophony” (DE 34). Dedicated leads, however, relied on intermediary organizations between the government and the public. These organizations included groups with regular contact with the public (e.g., health care professionals, community-based groups and leaders, and the media), which were perceived as crucial for engaging in two-way communication with the public and vulnerable groups through multiple channels and modes of communication that had—at least in part—been established prior to the pandemic.

Respondents further acknowledged the importance of trusted media channels, including social media, and the need to closely liaise with journalists and other media representatives in order to avoid misinformation, rumors, unintended reactions, or public resistance. In Germany the media strategy of the ERC lead was noted to be largely focused on press conferences, reporting COVID-19 infection numbers and scientific debates. In Singapore, Nigeria and—to some extent—Guinea, the media seemed to be strategically considered and media communication, including the use of different media and channels, was more integrated into the overall ERC strategy. This more comprehensive approach to media communication was evidenced by the media's use as a transmitter of coordinated communication, trainings of media representative on COVID-19, pro-bono time donated by key media, and explicit efforts of public and media representatives to collaborate in order to develop target-specific messages and disseminate them through appropriate channels.

While the necessity of collaborating with actors with direct contact to specific populations was recognized in all countries, public engagement similarly varied considerably across countries, ranging from systematic participatory approaches which drew on existing relationships in Guinea and Nigeria to more *ad-hoc*, informal attempts to engage with selected organizations and individuals when deemed necessary in Germany and Singapore. Especially in Germany and Singapore, respondents recalled situations where a top-down approach was pursued with governmental actors enlisting intermediaries as mouthpieces in their ERC strategy and expecting them to communicate messages and enforce measures. Interviewees highlighted that this led to difficulties as intermediaries had to communicate messages that they did not fully agree with or understand. It was also stressed that fulfilling intermediary roles required the use of considerable resources and funding that rarely seemed to be available to the organizations that took up these roles, and that these actors frequently mobilized their own resources due to a sense of responsibility and an altruistic, ideological self-concept rather than due to being included in the development of relevant ERC activities and messages.

On the other hand, the data show that the exchange of information and collaboration between a multitude of actors helped to leverage existing capacities and structures, enhance collective ownership of the messages, avoid public confusion and duplication of resources, and create a synergy of actions and ownership. Interviewees reported that the multisectoral nature of the pandemic response, and the large number of actors involved meant that complex processes were needed to develop, disseminate, and align messages, often resulting in considerable resources spent on reaching consensus. A Guinean public health authority representative, for example, described the substantial communication that was required to coordinate actors and make sure that duplication and ambiguity were avoided and new messages and activities were integrated into the overall ERC response:

“As soon as a partner specialized in the field of communication and community involvement […] contacts me, so I try to present him what we have already done, what we are doing in order to see how to integrate it in his systems. […] A criterion was put in place for the validation of messages so that the message would be unique, to avoid UNICEF communicating separately, WHO communicating separately, ANSS communicating separately.” (GI 01)

Despite tying considerable resources, good collaboration and coordination was perceived as crucial for managing the pandemic, whereas lack of coordination was criticized for jeopardizing the pandemic response.

### 3.4. Context

Contextual factors that were perceived as contributing to the success or failure of ERC related to the integration of ERC into the overall response, the direction of communication, the divergence of ERC strategies and messages, and the integration of lessons learned from previous outbreaks or other public health crises into the strategy.

#### 3.4.1. Integration of ERC into the emergency response

ERC was acknowledged to play a crucial role in the overall pandemic response and ideally integrated in the overall response through dedicated organizational structures right from the outset of the response. As illustrated by a Nigerian media communication specialist:

“Risk communication in terms of outbreak should no longer be like an afterthought. It has to be before outbreak, during outbreak, after outbreak, […] risk communication shouldn't be approached as an emergency step.” (NI 03)

#### 3.4.2. Direction of communication and community engagement

Several respondents acknowledged that the involvement of communities in developing an ERC strategy was an important factor for ensuring messages were adequately tailored for specific target groups, adapted locally, and accepted and owned by communities. Despite acknowledging the importance of community engagement, respondents reported that ERC in the first stages of COVID-19 had largely followed a top-down approach. They reported that such approach had been the preferred option for decision makers who, confronted with the fast development of the pandemic and a perceived lack of public knowledge about the disease, had felt that strong guidance had to be given quickly. By doing so, they had failed to consider the negative consequences of not including the public or specific community or target groups in the ERC development process from the start. Representatives of community groups and respondents who regularly engaged with citizens or patients, particularly in Germany and Singapore, reported that their engagement had been, for the most part, reactionary and *ad-hoc*. They also critiqued that they had little influence on ERC strategies, including message content, which frequently caused problems as they felt under pressure to communicate information that they either did not understand or did not agree with. In a few instances, respondents recalled that communication and engagement had been initiated by communities through a bottom-up approach (e.g., shopping for the elderly in Germany, providing handwashing buckets in public markets in Guinea), and that some of these initiatives had been taken up by the government and included in ERC strategies after proving effective.

#### 3.4.3. Divergence

Divergence of messages and pandemic response measures was reported as a major threat to ERC success, particularly as information and scientific evidence was noted to be in a continual flux. Divergence was a particular problem in governmental set-ups at national and regional levels (i.e., Germany, Nigeria and in part Guinea), whereas it was less of an issue in centrally governed Singapore. In Germany and Nigeria, the decision-making power and autonomy of the federal states with regards to the pandemic response made it challenging to coordinate decisions and align ERC. Respondents at local levels highlighted that they diverged from the goal of having a uniform country-wide pandemic response because of a perceived need to adapt strategies to the local situation, the availability of resources and the needs of local populations. In Nigeria, a prominent topic of discussion was the lack of resources and capacities at federal and local levels which hampered the government's ability to “effectively control the risk communication activities that were taking place” (representative of Nigerian Ministry of Health—NI 07).

#### 3.4.4. Learning from previous pandemic and risk communication experience

Some respondents reported drawing on their experiences of previous outbreaks and risk communication efforts and structures, particularly in Nigeria and Guinea, with Ebola, Lassa Fever, Cholera and Polio being cited as prominent examples. Previous experience meant that actors were more aware of the need to prepare an ERC strategy as early as possible, implement ERC structures, coordinate activities and messages, and respond to public reactions. A representative of an international organization working in Guinea referred to their experience during Ebola:

“We will remember that in Ebola's time, it was the bad communication that brought the reluctance. Well I, having been involved in Ebola with other people, we said to each other, we have to frame things not to give the floor to everyone. And those who have to intervene have to know what to do, what not to do, what not to say, so as not to fall into the same crisis situation as the crisis during Ebola” (GI 03).

In contrast, in Germany and, in part, Singapore, respondents reported a feeling that knowledge about handling PHE had been scarce and that ERC experience from previous crises or health problems had not been harnessed. A German senior infectious disease doctor voiced disappointment about how little knowledge had been applied from previous experiences:

“What reminded me a lot personally is the communication about HIV. […] So, I think I noticed many elements in risk communication that are well known. […] I was also shocked by how little has been learned from this communication.” (DE 11)

## 4. Discussion

Germany, Guinea, Nigeria, and Singapore launched extensive ERC once the first COVID-19 case was identified and the importance of mitigating COVID-19 was undisputed. From these four countries, we identified a number of mechanisms that can facilitate ERC during PHE, many of which are in line with WHO risk communication guidelines, including on (i) building trust and engaging communities, (ii) integrating ERC into health and emergency response systems, and (iii) ERC practice (3). In Box 1, we provide some brief recommendations with regard to the three topics. Below, we elaborate on each point in more detail.

Box 1 **Key recommendations for Emergency Risk Communication in Public Health Emergencies.**

**Building trust and engaging communities**

- Routinely map, and build long-term relationships with, stakeholders who you will work with during a PHE.
- Closely collaborate with those who directly interact with communities, including health professionals and civil society, in developing and implementing the ERC strategy.
- Ensure that stakeholders who implement ERC are supported.

**Integrating ERC into health and emergency response systems**

- Simultaneous plan preventive and communicative measures and revise ERC strategies regularly in accordance with the pandemic response.
- Prioritize coordination in order to avoid public confusion, frustration, and lack of compliance.

**ERC practice**

- Prioritize two-way communication in order to respond to public concerns, develop tailored communication and empower communities.
- Embrace social media communication as a core component of ERC.

Trust and community engagement are key recommendations of the WHO guidelines. Accordingly, our analysis shows that good ERC depends on good relationships with those working and interacting closely with communities, like health professionals and civil society organizations. ERC strategy do not only need to account for the collaboration of lead actors involved in ERC development and implementation but also for the early engagement of those with direct contact to local communities and their representatives (3). A prerequisite for good collaboration includes routine mapping, identification and engagement of actors across levels. By doing this, established organizational and communication structures can be leveraged in order to quickly initiate ERC and reach priority or vulnerable groups (2). The analysis also shows that when working with local communities and their representatives, it is important to provide financial and other forms of support in order to not over-burden existing structures and capacities.

WHO acknowledges the need to integrate ERC into the overall PHE response, a recommendation which is strongly mirrored in the data presented above. Our analysis shows that the firm integration of ERC into an overall outbreak response, prior to and at the early onset, is key to responding to PHE. This integration is ideally achieved through simultaneous planning of preventive and communicative measures and through the regular revision of ERC strategies across levels (16, 34). Employing ERC-specific expertise and resources can ensure that up-to-date knowledge on communication and its impact is applied, strategies are devised in a way that is realistic, and best practice is implemented. Our analysis shows that ERC is often not given the same resources or ideological value as other emergency response components, highlighting an urgent need for higher prioritization of ERC within overall response structures (3).

In line with WHO's recommendation on governance and information structures, our study shows that collaborative and centrally coordinated governing structures are conducive to consistent and cohesive ERC, whereas lack of collaboration and coordination increases the risk of parallel structures, duplication of resources and cacophony of messages (3). This in turn almost inevitably results in public confusion, frustration, and lack of compliance (10, 35–37). Collaboration and coordination are even more important within federal or decentralized systems where a multitude of actors must be coordinated and challenges concerning information sharing, activity coordination, divergence and inefficiency have to be overcome (10, 16, 38). Our findings complement previous literature and WHO IHR core capacity indicators which show that defining leadership, assigning responsibilities, and developing protocols for collaboration enables governments to quickly respond to emerging PHE (2, 3, 34).

Finally, actual ERC practice, including strategic planning, monitoring and evaluation, message design and communication channels is identified as a key feature of ERC, underpinning WHO's recommendations in this area. Our study puts a particularly strong focus on two-way communication as a crucial, yet largely neglected, component of ERC during COVID-19. The fact that two-way communication was often not established or insufficiently used resulted in a failure to leverage the potential to respond to public concerns, tailor messages, and address rumors and misinformation. Our findings align with existing literature which suggests that in times of insecurity and uncertainty, key ERC actors tend to employ authoritative, one-way communication instead of responding to people's needs for two-way communication (10). Previous research shows that a lack of two-way communication can lead to decreased public trust which, in turn, is a barrier to public uptake of prevention measures (3, 10, 17, 39–42). Highlighting a lack of bilateral communication during COVID-19, Dickman and Strahwald call for a new understanding of ERC which moves away from top-down ERC approaches and toward participatory approaches that foster community empowerment. Our analysis suggests that Nigeria and Guinea were more likely to implement a participatory approach and incorporate the views of community leaders and organizations with direct public contact in their ERC strategies than Singapore and Germany. Such differences suggest that countries in the global north could learn from countries in the global south which have more experience in responding to pandemics and communicating in PHE.

The analysis further suggests that social media is an important component of ERC, but that the way in which it is used determines the usefulness. Our findings indicate that the dissemination of messages through social media is by now routine practice in ERC. Yet, in some cases (e.g., in Germany), social media was mainly used as an additional mode of communication and that its added value was limited because its potential was not fully seized. In other cases (e.g., in Singapore and Guinea), however, social media were used to engage specific target audiences, track rumors and monitor and respond to online discussions. Respondents highlighted that used in this way, social media communication formed an indispensable part of ERC. Our findings suggest that modern ERC strategies need to embrace unregulated, informal, community-level, and multi-channel communication as opportunities which should be seized rather than as threats that have to be contained. Bilateral communication needs to become an integral part of ERC in order to maximize its impact (3, 4).

A number of limitations deserve acknowledgment. First, this study only captures information on the early phases of ERC and does not include information on later stages of the pandemic. Second, while our paper identifies the overarching structural factors of importance for ERC during PHE, it also highlights that local contextual factors play an important role. Third, the direct effect of ERC policies across countries on behavior lacks distinct measurement indicators. Fourth, given that in each country, with the exception of Singapore, a selection of regional states was sampled, the findings cannot be assumed to represent all states' governing structures. Finally, some key informants were unavailable for an interview or had recently changed roles. The semi-structured nature of the interviews, incorporation of multiple types and levels of actors and triangulation with the document review, however, provided ample opportunity to reduce respondent bias, contextualize the data, and develop an in-depth understanding of similarities and differences in ERC strategies.

## 5. Conclusion

The COVID-19 pandemic has provided several key lessons for enacting and enabling effective ERC during PHE. It emphasizes the need to amend ERC strategies according to the progression, response, and public's reaction to the pandemic. While our analysis indicates that the nature of the pandemic brought a set of unexpected challenges and learning opportunities, it also highlights that drawing on experience and expertise from previous outbreaks allowed countries to quickly activate and adapt ERC. This suggests that countries with little outbreak experience can learn from countries with more extensive experience. Strengthening the incorporation of ERC into emergency response plans, actively building networks and relationships between relevant stakeholders outside of emergency times, and incorporating community leaders and bilateral communication into ERC governing structures are key to successful ERC in future outbreaks.

## Data availability statement

The datasets presented in this article are not readily available because due to the qualitative nature of the data, the confidentiality agreements signed and the ease with which respondents might be identified based on the content of the transcripts, we are unable to make the interview transcripts publicly available. A copy of the consent form can be requested from the corresponding author. Requests to access the datasets should be directed to el-bcheraouiC@rki.de.

## Ethics statement

The studies involving human participants were reviewed and approved by Charité—Universitätsmedizin Berlin (EA2/148/20), the Comité National D'Ethique pour la Recherche en Santé (105/CNERS/20) in Guinea, the National Health Research Ethics Committee of Nigeria (NHREC/01/01/2007-19/08/2020), and the National University Singapore Institutional Review Board (NUS-IRB-2020-434). The patients/participants provided their written informed consent to participate in this study.

## Author contributions

BG and HW wrote the original draft and visualized the data. CE, AM, and EB conceptualized the study and acquired funding. BG, FC, CR, KA, MT, LB, and PT curated the data. BG, HW, AM, FC, CR, KA, MT, BS, LB, PT, and JB analyzed the data. BG, HW, AM, FC, CR, KA, MT, PT, JB, TB, EB, SA-A, and CE collected the data. SA-A, AD, AJ, HL-Q, and CE administered the project. BG, FC, CR, KA, MT, BS, and SA-A were responsible for the provision of resources and software. AD, AJ, HL-Q, HW, AM, and CE supervised the study. HW, AM, and CE were responsible for the validation of the results. All authors wrote, reviewed, edited the manuscript, and were involved in the design of the study and its methodology.
