# Supplementary material for: Communicating risk during early phases of COVID-19: Comparing governing structures for emergency risk communication across four contexts
Source: Front Public Health. 2023 Jan 27;11:1038989. doi: 10.3389/fpubh.2023.1038989 (PMC9911432; doi:10.3389/fpubh.2023.1038989)
Supplement: Supplementary file 1 [file Data_Sheet_1.docx]

**Supplementary Box: Description of national policies outlining ERC**

*Germany:* In Germany, the National Public Health Institute’s *National Pandemic Plan* (Part I and Part II) forms the basis for planning, preparing, and responding to pandemics.^1,2^ Part I describes the necessary structures and measures for planning and responding to an influenza pandemic. Part II provides an overview of the scientific evidence base, standard pharmaceutical and non-pharmaceutical interventions, and other relevant topics to be considered. The document identifies the Ministry of Health as the central coordinating ministry, while recognising the specific roles of other ministries, subordinate institutes and interest groups. Both documents acknowledge the importance of early risk communication and public engagement activities in a pandemic and outline strategic points for planning, designing and coordinating related activities. In March 2020, a *COVID-specific supplement to the National Pandemic Plan* was published which confirms the Ministry of Health as the ERC strategy lead. The supplement specifies communication roles and channels by institute competency and target groups.^3^ The National Pandemic Plan and the added supplement form the guidance for localized ERC plans at the level of federal state health authorities and local public health offices.^4^ Each federal state has its own influenza pandemic plan which varies in depth, coordination, responsibilities, and authority. Strategies and measures are adapted locally.

*Guinea:* The *Annual plan of communication, social mobilization and community engagement against COVID-19 in Guinea*^5^ describes the plan of action of the Ministry of Health of Guinea for communication, social mobilization, and community engagement for 2020 to fight COVID-19 at national, regional and community levels. The plan outlines key actors as well as specific objectives and activities. The plan assigns the National Health Security Agency as the designated lead organisation and coordinator of the response, while regional health offices are assigned responsibility for implementation and monitoring. UNICEF is specifically named as providing technical support and overall oversight. The plan puts particular emphasis on mobilising populations, building risk communication and community engagement capacity at all levels through trainings, and providing vulnerable communities with hygiene materials in order to facilitate participation in prevention measures.

*Nigeria:* In Nigeria, the *National COVID-19 Pandemic Multi-Sectorial Response Plan* provides “a blueprint for a coordinated national strategy to respond to the COVID-19 pandemic".^6^ Published in May 2020 by the Presidential Task Force and updated several times, the document outlines the structure and stakeholders of the risk communication and community engagement (RCCE) pillar as an integral part of the response ^6^. The additional *Risk Communication and Community Engagement Strategy* further outlines key aspects of the strategy in detail, including the rationale, implementation framework with the public, stakeholders and communities, capacity building, rumour management, and a monitoring and evaluation plan.^7^ In addition, the National Public Health Care Development Agency on Preparedness & Response to Covid-19 sets out a strategy on developing and implementing “a robust COVID-19 risk communication plan” for primary health care.^8^ The above documents are complemented by the National and State training of trainers on RCCE which lays out how ERC should be implemented at the federal state level.^9^

*Singapore:* The main national strategic document for public health emergencies is the *Ministry of Health pandemic readiness and response plan for influenza and other acute respiratory diseases* issued in 2014.^10^ It outlines and recommends public health measures and actions that can guide implementation during a pandemic. In terms of communication, the plan lists the Ministry of Health as a key communicator during a pandemic. Accordingly, the Ministry of Health pandemic readiness and response plan for influenza and other acute respiratory diseases was complemented during COVID-19 by the government’s regular updates through media, press releases, and ministerial statements which outlined the response actions according to the severity of the situation and provided an indication of stakeholder engagement during the pandemic. Individual healthcare and non-healthcare agencies were able to develop individual communication plans considering the infection control guidelines provided by the Ministry of Health plan.

**References**

1. Robert Koch Institute. *Nationaler Pandemieplan Teil I*. Internet. 2017. *Strukturen und Maßnahmen*. Accessed 2021 June 30. <https://edoc.rki.de/bitstream/handle/176904/187/28Zz7BQWW2582iZMQ.pdf?sequence=1&isAllowed=y>

2. Robert Koch Institute. *Nationaler Pandemieplan Teil II*. Internet. 2016. *Wissenschafliche Grundlagen*. Accessed 2021 June 30. <https://www.rki.de/DE/Content/InfAZ/I/Influenza/Pandemieplanung/Downloads/Pandemieplan_Teil_II_gesamt.pdf?__blob=publicationFile>

3. Robert Koch Institute. *Ergänzung zum Nationalen Pandemieplan – COVID-19 – neuartige Coronaviruserkrankung*. Internet. 2020. 2020 March 4. Accessed 2021 March 15. <https://www.rki.de/DE/Content/InfAZ/N/Neuartiges_Coronavirus/Ergaenzung_Pandemieplan_Covid.html>

4. Daten für Taten – „Gesundheitsmonitoring für evidenzbasiertes Handeln“ ist das Motto für den Tag des Gesundheitsamtes 2020. 13 March, 2020. <https://www.rki.de/DE/Content/Service/Presse/Pressemitteilungen/2020/03_2020.html>

5. Ministère de la Santé, Agence Nationale de Sécurité Sanitaire. Plan annuel de communication, de mobilisation sociale et d’engagement communautaire contre le COVID 19 en Guineee. 2020.

6. Nigerian Presidential Task Force for the Control of Coronavirus (COVID-19). National COVID-19 Pandemic Multi-Sectoral Response Plan. Federal Government of Nigeria. March 30, 2021. Updated 2020 August 14. Accessed March 30, 2021. <https://statehouse.gov.ng/covid19/wp-content/uploads/2020/09/National-COVID-19-Multi-Sectoral-Pandemic-Response-Plan_Post-MTR_September-2020.pdf>

7. Federal Government of Nigeria, Nigeria Center for Disease Control, Nigerian Presidential Task Force on COVID-19. *Risk Communicationand Community Engagement Strategy COVID-19. Prevention and Control in Nigeria*. Vol. 2021. 2020. Accessed 2021 March 30. <https://covid19.ncdc.gov.ng/media/files/UPDATED_RCCE_Strategy_web_version.pdf>

8. Nigerian National Public Health Care Development Agency. Preparedness and Response to Coronavirus Disease 2019 (Covid-19) at Primary Healthcare and Community Level. National Public Health Care Development Agency. Accessed March 30, 2021. <https://www.alnap.org/system/files/content/resource/files/main/guide_on_phc_preparedness_and_response-covid-19.pdf>

9. Federal Ministry of Health, Nigeria Centre for Disease Control. Training Of Trainers On Integrated Risk Communication And Community Engagement For Covid-19 Response In Nigeria. Abudja2020.

10. Ministry of Health Singapore. Being Prepared for a Pandemic Singapore. Accessed April 16, 2021. <https://www.moh.gov.sg/diseases-updates/being-prepared-for-a-pandemic>
